# miRNA Regulatory Networks Associated with Peripheral Vascular Diseases

**DOI:** 10.3390/jcm11123470

**Published:** 2022-06-16

**Authors:** Daniel P. Zalewski, Karol P. Ruszel, Andrzej Stępniewski, Dariusz Gałkowski, Marcin Feldo, Janusz Kocki, Anna Bogucka-Kocka

**Affiliations:** 1 Chair and Department of Biology and Genetics, Medical University of Lublin, 4a Chodźki St., 20-093 Lublin, Poland; anna.kocka@umlub.pl; 2 Department of Clinical Genetics, Chair of Medical Genetics, Medical University of Lublin, 11 Radziwiłłowska St., 20-080 Lublin, Poland; karol.ruszel@umlub.pl (K.P.R.); janusz.kocki@umlub.pl (J.K.); 3 Ecotech Complex Analytical and Programme Centre for Advanced Environmentally Friendly Technologies, University of Marie Curie-Skłodowska, 39 Głęboka St., 20-612 Lublin, Poland; andrzej.stepniewski@umcs.pl; 4 Department of Pathology and Laboratory Medicine, Rutgers-Robert Wood Johnson Medical School, One Robert Wood Johnson Place, New Brunswick, NJ 08903-0019, USA; galkowd@fastmail.fm; 5 Chair and Department of Vascular Surgery and Angiology, Medical University of Lublin, 11 Staszica St., 20-081 Lublin, Poland; martinf@interia.pl

**Keywords:** miRNA, lower extremity artery disease, chronic venous disease, abdominal aortic aneurysm, miRNA expression, next-generation sequencing

## Abstract

A growing body of evidence indicates a crucial role of miRNA regulatory function in a variety of mechanisms that contribute to the development of diseases. In our previous work, alterations in miRNA expression levels and targeted genes were shown in peripheral blood mononuclear cells (PBMCs) from patients with lower extremity artery disease (LEAD), abdominal aortic aneurysm (AAA), and chronic venous disease (CVD) in comparison with healthy controls. In this paper, previously obtained miRNA expression profiles were compared between the LEAD, AAA, and CVD groups to find either similarities or differences within the studied diseases. Differentially expressed miRNAs were identified using the DESeq2 method implemented in the R programming software. Pairwise comparisons (LEAD vs. AAA, LEAD vs. CVD, and AAA vs. CVD) were performed and revealed 10, 8, and 17 differentially expressed miRNA transcripts, respectively. The functional analysis of the obtained miRNAs was conducted using the miRNet 2.0 online tool and disclosed associations with inflammation and cellular differentiation, motility, and death. The miRNet 2.0 tool was also used to identify regulatory interactions between dysregulated miRNAs and target genes in patients with LEAD, AAA, and CVD. The presented research provides new information about similarities and differences in the miRNA-dependent regulatory mechanisms involved in the pathogenesis of LEAD, AAA, and CVD.

## 1. Introduction

Permanent progress in the development of research methods allows the elucidation of molecular mechanisms involved in the pathogenesis of human diseases. One of the most intensively studied aspects in this field is the role of the microRNA (miRNA) regulatory function in the processes underlying pathological conditions.

miRNAs constitute a group of non-coding and single-stranded RNA molecules with a length of 18–25 nucleotides. The fundamental role of miRNA is post-transcriptional regulation of gene expression through direct binding to messenger RNA (mRNA) strands, causing mRNA degradation or inhibition of translation [1,2]. Many previous studies have indicated altered expression of miRNAs in patients with various diseases, including cancer, autoimmunology, infections, preeclampsia, and cardiovascular diseases, indicating a potential utility of miRNAs in diagnosis and therapy [2,3,4,5].

Our research group is focused on the effect of alterations in the miRNA–gene regulatory network on the pathogenesis of three vascular diseases: lower extremity artery disease (LEAD), abdominal aortic aneurysm (AAA), and chronic venous disease (CVD). LEAD is caused by the chronic development of atherosclerotic plaques in the arteries of the lower extremities, leading to ischemic events, including intermittent claudication and critical limb ischemia [6,7,8,9,10]. The development of AAA is related to a focal dilatation of the abdominal aorta measuring 50% greater than the normal proximal segment or >3 cm in maximum diameter. The progression of AAA can lead to aneurysm rupture, an event that reaches 60–80% of mortality [11,12,13,14,15]. In turn, CVD is defined as a syndrome of chronic morphological and functional abnormalities in the venous circulation of the lower extremities, resulting from venous valve incompetence, venous reflux, and venous hypertension [16,17,18,19,20,21,22,23,24]. Despite the different clinical images of these diseases, they share many common pathological conditions, including endothelial dysfunction, inflammatory response, and vascular remodeling [6,7,11,12,16,17,20]. Furthermore, the high prevalence, multifactorial character, and burden of severe life-threatening consequences of these diseases made them major global health problems [6,8,9,10,13,14,15,18,19,22,24]. Therefore, intensive research is desired to propose new and more effective strategies for the diagnosis and therapy of LEAD, AAA, and CVD.

Many studies have indicated an important role of miRNAs in vascular pathology [25,26,27,28] and have pointed to certain dysregulated miRNAs as candidates for biomarkers of LEAD [29,30], AAA [31], and CVD [32,33,34]. Special attention is paid to the identification of circulatory miRNA biomarkers (from whole blood, blood cells, plasma, or serum) with potential utility as diagnostic, prognostic, and therapeutic targets for these diseases [35,36,37,38,39,40].

In our previous studies, we presented an integrated analysis of miRNA and gene expression in peripheral blood mononuclear cells (PBMCs) of patients with LEAD, AAA, and CVD in comparison with control subjects to identify alterations in miRNA regulatory function associated with these diseases [40,41,42]. However, it is unknown whether the miRNA expression profiles differ between the analyzed diseases. Therefore, in this paper, we present the results of the next step of our study, which includes a pairwise comparative analysis among the LEAD, AAA, and CVD groups, together with the functional analysis and identification of potential targets of dysregulated miRNAs. Identification of similarities and differences in the expression of the miRNAs in the regulatory network could provide a deeper insight into the vascular pathology underlying these diseases. Identification of disease-specific biomarkers could potentially enable the development of new methods for diagnosis and treatment.

## 2. Materials and Methods

In our previous studies, miRNA expression profiles of PBMCs were compared between patients with vascular diseases (LEAD, AAA, and CVD) and healthy controls [40,41,42]. For a deeper characterization of the molecular mechanisms involved in the pathogenesis of LEAD, AAA, and CVD, we performed and presented in this paper the next step of the study, where miRNA expression profiles were compared between the studied diseases using the following comparisons: LEAD vs. AAA, LEAD vs. CVD, and AAA vs. CVD.

### 2.1. Study Participants

The study was carried out in accordance with the Declaration of Helsinki and after the approval of the Ethics Committee of the Medical University of Lublin (decision No. KE-0254/341/2015). The study group consisted of 40 patients with LEAD, 28 patients with AAA, and 34 patients with CVD. The clinical examination of the patients was carried out at the Independent Public Clinical Hospital No. 1 in Lublin. Informed and signed consent was obtained from all study subjects.

Patients with LEAD were evaluated using peripheral pulse examination, ankle-brachial index test, treadmill test, angiography, and duplex color flow ultrasound scanning. Individuals diagnosed with intermittent ischemia-associated claudication without critical ischemia (Trans-Atlantic Inter-Society Consensus score B or C and Rutherford category 2 or 3) and with atherosclerotic lesions localized in the femoral, iliac, or popliteal arteries were included. Patients with AAA were diagnosed using duplex ultrasonography and contrast-enhanced spiral computed tomography with volume-rendered reconstructions. AAA patients with a maximum diameter of the aneurysm ranging from 5.6 to 7.8 cm (mean = 6.39, standard deviation = 0.633) were included. CVD patients were examined using tourniquet test, auscultation, and duplex ultrasound scanning, and those who had symptoms classified according to CEAP as varicose veins (C2) of superficial veins (As) with primary etiology (Ep) and reflux pathophysiology (Pr) were included. Established exclusion criteria common to all studied diseases include type 1 diabetes mellitus, previous vascular surgery, inflammatory diseases, and pregnancy. A detailed presentation of the clinical characteristics of the participants and a full description of the inclusion and exclusion criteria are provided in our previous articles [40,41,42].

### 2.2. miRNA Expression Datasets

The miRNA expression datasets were generated by RNA sequencing of the PBMC samples obtained from the study participants as described in our previous papers [40,41,42]. Briefly, PBMC specimens were isolated from whole blood samples using density gradient centrifugation with Gradisol L reagent (Aqua-Med, Łódź, Poland). A diversity of white blood cell subpopulations in the studied groups was evaluated using laboratory analysis of blood morphology (Figure S1). Small RNA fractions were isolated from PBMC samples using the MirVana microRNA Isolation Kit (Ambion, Austin, TX, USA). The quantity and quality of the obtained small RNA samples were assessed using the Agilent 2100 Bioanalyzer (Agilent Small RNA Kit, Agilent Technologies, Santa Clara, CA, USA). Small RNA samples were subjected to preparation of miRNA libraries using the Ion Total RNA-Seq Kit v2, Magnetic Bead Cleanup Module kit, and Ion Xpress RNA-Seq Barcode 01-16 Kit (Life Technologies, Carlsbad, CA, USA). Libraries were sequenced on the Ion 540 chips (Life Technologies) using the Ion S5 XL System (Thermo Fisher Scientific, Waltham, MA, USA). Raw sequences were aligned to 2792 human miRNAs from miRBase v21 (http://www.mirbase.org) using the Torrent Suite Software v5.0.4. and the Ion Torrent Small RNA Plugin v5.0.5r3 plugin (Thermo Fisher Scientific, Waltham, MA, USA). The parameters describing miRNA libraries and the primary results of the sequencing data analysis are provided in the supplementary materials provided together with our previous publications [40,41,42].

### 2.3. Data Analysis

Data analysis was performed using the R environment (version 4.2.0, https://www.r-project.org) and the appropriate packages according to the corresponding reference manuals. 

The statistical significance of the differences in demographic and clinical parameters between the LEAD, AAA, and CVD groups was examined using the Kruskal–Wallis rank sum test for continuous variables (kruskal.test function in R) and the two-sided Fisher’s exact test for categorical variables (fisher.test function in R).

All other statistical procedures applied to expression datasets and subsequent bioinformatic analysis were previously described in detail in [40,41,42]. The uniformity of the expression data was confirmed on the boxplot of Cook’s distances of miRNAs across all analyzed samples (Figure S2). The quality of differential expression analysis results was assessed on MA plots and histograms of the *p*-values obtained for each comparison (Figures S3–S5). Differential expression analysis of expression datasets was performed on biological replicates using the DESeq2 method implemented in the DESeq2 1.36.0 package [43] (https://bioconductor.org/packages/release/bioc/html/DESeq2.html (accessed on 28 March 2022)).

DESeq2 analysis was performed on expression data filtered out of genes with a mean of reads number lower than 1. The analysis was carried out in multivariate mode, using disease status as a variable of interest and the following characteristics as covariates: age; body mass index; morphology test results (hematocrit and counts of monocytes, eosinophils, basophils, and platelets); creatinine levels; sex; smoking (never and former smokers vs. current smokers); hypertension; type 2 diabetes; coronary artery disease; and medication with statins, acetylsalicylic acid, clopidogrel, beta-adrenergic blockers, angiotensin-converting enzyme inhibitors, Ca^2+^ channel blockers, fibrates, micronized diosmin, and preparation containing hesperidin, *Ruscus aculeatus*, and vitamin C. Differentially expressed miRNAs received from the DESeq2 analysis with *p*-value below 0.05, absolute log2 fold change > 1, and mean of normalized counts >10 were selected. 

Venn diagrams and heatmap with hierarchical clustering were created using VennDiagram 1.7.3 (https://cran.r-project.org/web/packages/VennDiagram/index.html (accessed on 28 March 2022)) [44] and pheatmap 1.0.12 (https://cran.r-project.org/web/packages/pheatmap/index.html (accessed on 28 March 2022)) packages, respectively.

A receiver operating characteristic (ROC) analysis implemented in the pROC 1.18.0 package [45] (https://cran.r-project.org/web/packages/pROC/index.html (accessed on 28 March 2022)) was used to evaluate the classification performance of selected miRNAs. For ROC analysis and visualizations, filtered expression data were transformed using regularized log normalization (the rlog function in DESeq2 package).

The biological role of selected miRNAs was explored using the miRNet 2.0 online platform [46] (https://www.mirnet.ca) using a hypergeometric test applied to functional terms of the ‘miRNA-Function’ category. The miRNet 2.0 database was also used to identify interactions between miRNAs selected in this study and genes selected in the previous study [47]. Obtained interactions formed a regulatory network, which was presented using Cytoscape v3.9.1 software (https://cytoscape.org/) [48].

The identification of relationships between the characteristics of the study participants and the expression of selected genes was performed using the Spearman rank correlation test implemented in the Hmisc 4.7-0. package (https://cran.r-project.org/web/packages/Hmisc/index.html (accessed on 28 March 2022)) as well as a two-sided Mann–Whitney *U* test implemented in wilcox.test function in R. Multivariate linear regression models were constructed using the lm() base function in R, and obtained results were summarized using sjPlot 2.8.10 package (https://cran.r-project.org/web/packages/sjPlot/index.html (accessed on 28 March 2022)).

## 3. Results

### 3.1. The Study Group Characteristics

The study group included 40 patients with LEAD, 28 patients with AAA, and 34 patients with CVD. Information on the characteristics of the participants was provided separately for each disease in our previous papers [40,41,42], and here it is summarized in Table 1. Statistically significant differences (*p* < 0.05) in some characteristics were disclosed between the LEAD, AAA, and CVD groups, especially in age, hypertension status, blood cell counts, creatinine serum level, and medication (Table 1). The observed differences were difficult to avoid during the construction of the study population because they reflect different risk factors, comorbidities, and medications related to particular diseases. The relationships between these factors and the expression of selected miRNAs are investigated in Section 3.5.

### 3.2. Identification of Differentially Expressed miRNAs between the LEAD, AAA, and CVD Groups

The miRNA expression profiles were compared between the studied diseases using the following comparisons: LEAD vs. AAA, LEAD vs. CVD, and AAA vs. CVD. The multivariate DESeq2 method was applied to select the most relevant differentially expressed miRNAs using disease status as a variable of interest and characteristics differentiating the study groups with a statistical significance of *p* < 0.05 (see Table 1). Application of established selection criteria allowed the selection of 10 miRNA transcripts (8 upregulated and 2 downregulated) from the LEAD vs. AAA comparison, 8 miRNA transcripts (4 upregulated and 4 downregulated) from the LEAD vs. CVD comparison, and 17 miRNA transcripts (9 upregulated and 8 downregulated) from the AAA vs. CVD comparisons (Table 2). The detailed results of the differential expression analysis concerning all miRNA transcripts are provided in Tables S1–S3.

**Figure 1:**
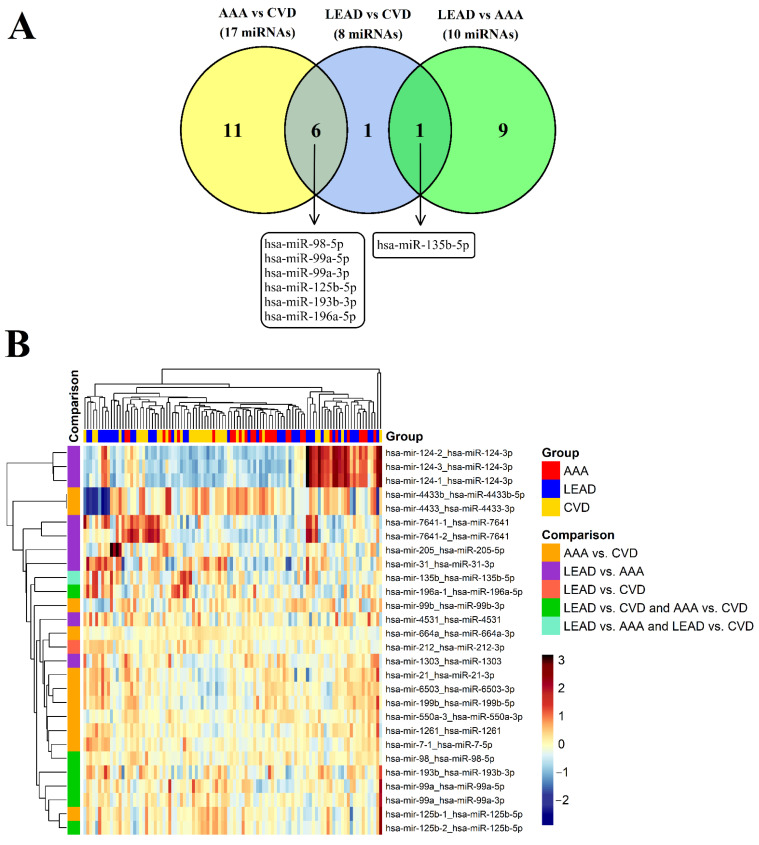
The comparison of the sets of differentially expressed miRNA transcripts selected from pairwise comparative analysis performed among the LEAD, AAA, and CVD groups using DESeq2 method. (**A**) The comparison of the sets of miRNA transcripts selected from LEAD vs. CVD, LEAD vs. AAA, and AAA vs. CVD comparisons using the following selection criteria: *p*-value < 0.05, absolute log2 fold change > 1, and mean of normalized counts > 10. The number in the intersection fields of the Venn diagram represents the number of miRNA transcripts common to the comparisons. The frames below the Venn diagram include six miRNAs common for AAA vs. CVD and LEAD vs. CVD comparisons and one miRNA common for LEAD vs. CVD and LEAD vs. AAA comparisons. (**B**) Heatmap of the expression of 28 miRNA transcripts selected from LEAD vs. AAA, LEAD vs. CVD, and AAA vs. CVD comparisons and included in the Venn diagram in panel A. Hierarchical clustering was performed using the average method applied to Euclidean distances. AAA—abdominal aortic aneurysm, CVD—chronic venous disease, LEAD—lower extremity artery disease.

To identify similarities and differences between the sets of miRNA transcripts selected from the performed comparisons, the sets of 10, 8, and 17 miRNA transcripts were compared in a Venn diagram (Figure 1A). Six miRNAs were common for the LEAD vs. CVD and AAA vs. CVD comparisons, and one miRNA, hsa-miR-135b-5p, was common for the LEAD vs. CVD and AAA vs. CVD comparisons (Figure 1A). The expression of selected miRNA transcripts was visualized on a heatmap with hierarchical clustering (Figure 1B).

The classification performance of selected miRNA transcripts was further evaluated using ROC analysis, which shows moderate classification ability (areas under the ROC curves ranged from 0.824 to 0.450) (Table 2). The detailed results of the ROC analysis are provided in Figures S6–S8 and Table S4.

### 3.3. Selected miRNAs Are Closely Related to Vascular Pathology

**Figure 2:**
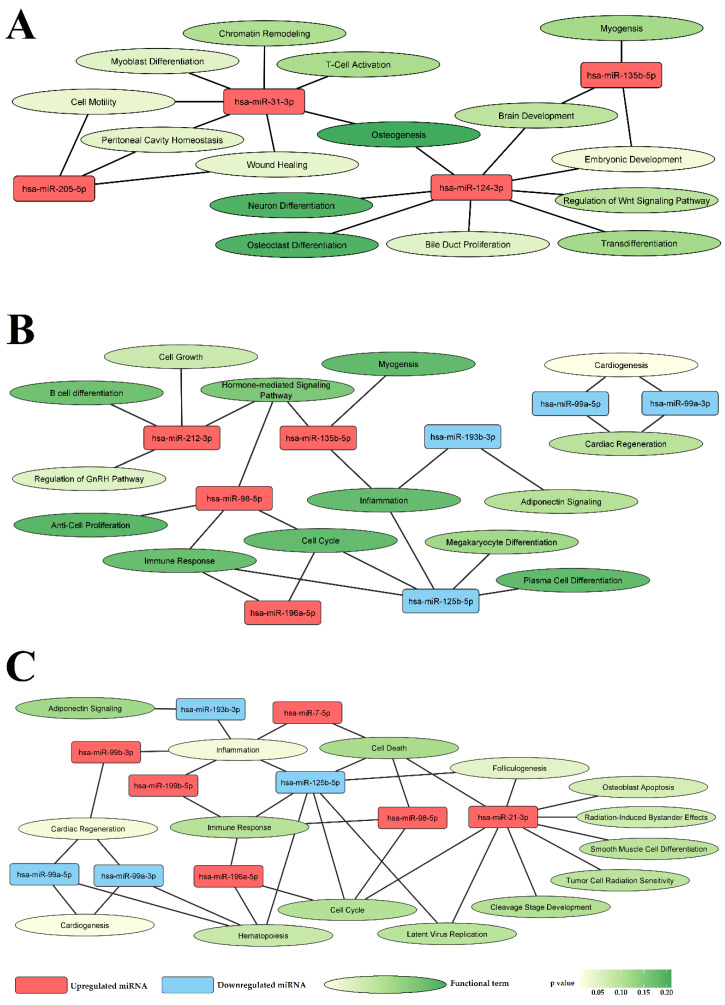
The functional network of miRNAs selected from pairwise comparisons among the LEAD, AAA, and CVD groups. (**A**) Network for miRNAs selected from the LEAD vs. AAA comparison. (**B**) Network for miRNAs selected from the LEAD vs. CVD comparison. (**C**) Network for miRNAs selected from the AAA vs. CVD comparison. Each panel presents the top 15 most enriched functional terms (with the lowest *p*-value of enrichment) of ‘miRNA Function’ category in the miRNet 2.0 tool as well as associated miRNAs. AAA—abdominal aortic aneurysm, CVD—chronic venous disease, LEAD—lower extremity artery disease.

The biological role of dysregulated miRNAs was explored using the miRNet 2.0 online platform (https://www.mirnet.ca). This tool requires mature miRNA IDs; therefore, the names of the miRNA transcripts were transformed into the corresponding mature miRNA IDs according to miRBase 22.1 (http://www.mirbase.org). Thus, functional analysis was performed for 7 unique mature miRNAs given by 10 miRNA transcripts selected from the LEAD vs. AAA comparison, 8 unique mature miRNAs given by 8 miRNA transcripts selected from the LEAD vs. CVD comparison, and 16 unique mature miRNAs given by 17 miRNA transcripts selected from the AAA vs. CVD comparison (Table 2). The functional analysis was performed using a hypergeometric test applied to functional terms of the ‘miRNA-Function’ category in the miRNet 2.0 tool. The top 15 most enriched functional terms (with the lowest *p*-value of enrichment) and associated miRNAs are presented in Figure 2 and Table S5. Analyzed miRNAs were associated with processes closely related to vascular pathology, including inflammation and cell differentiation, motility, and death (Figure 2).

### 3.4. Selected miRNAs Regulate Genes Differentially Expressed between the LEAD, AAA, and CVD Groups

To construct a miRNA–gene regulatory network for selected miRNAs, the results of this study were integrated with the results of our previous paper, in which the whole transcriptome expression profiles of LEAD, AAA, and CVD patients were analyzed and the sets of 21, 58, and 10 differentially expressed genes were revealed from the LEAD vs. AAA, LEAD vs. CVD, and AAA vs. CVD comparisons, respectively [47]. The altered expression of these genes could be an effect of the dysregulation of miRNAs found in the current study. To examine this effect, the sets of miRNAs selected in this study were subjected to the miRNet 2.0 tool to identify the targeted genes, and then the received sets of targets were searched for differentially expressed genes found in our previous work.

The identification of targets for seven miRNAs selected from the LEAD vs. AAA comparison revealed a set of 7359 genes, containing 3 genes (*GIT2*, *UFM1*, and *YBX1*) previously shown to be dysregulated between the LEAD and AAA groups. The identification of targets for eight miRNAs selected from the LEAD vs. CVD comparison disclosed a group of 5980 genes, containing 8 genes (*ARL6IP1*, *C1orf216, DNAH1, EIF3C*, *FAM167A*, *HECTD4*, *PSME1*, and *TSC2*) previously shown to be dysregulated between the LEAD and CVD groups. The identification of targets for 16 miRNAs selected from the AAA vs. CVD comparison disclosed a set of 8006 genes, containing 2 genes (*MALT1* and *STMN3*) previously shown to be dysregulated between the AAA and CVD groups. Detailed information regarding identified interactions is provided in Table S6.

**Figure 3:**
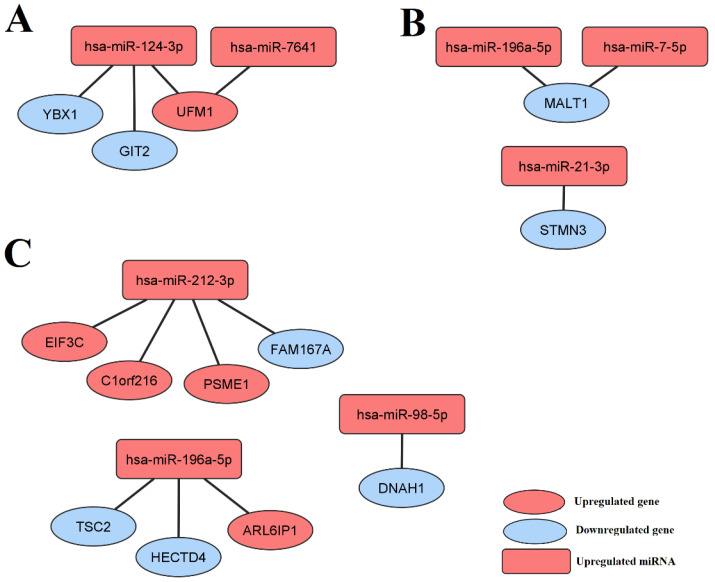
The regulatory network containing miRNAs selected in the current study and genes selected from a previous study [47] regarding (**A**) LEAD vs. AAA comparison, (**B**) AAA vs. CVD comparison, and (**C**) LEAD vs. CVD comparison. AAA—abdominal aortic aneurysm, CVD—chronic venous disease, LEAD—lower extremity artery disease.

The interactions found between miRNAs selected in this study and genes reported in the previous study were visualized on regulatory networks, including four unique miRNA–gene pairs between two miRNAs and three genes resulting from the LEAD vs. AAA comparison, eight unique miRNA–gene pairs between three miRNAs and eight genes resulting from the LEAD vs. CVD comparison, and three miRNA–gene pairs between three miRNAs and two genes resulting from the AAA vs. CVD comparison (Figure 3).

### 3.5. Some of the Selected miRNAs Are Associated with the Clinical and Demographic Characteristics of the Study Groups

A correlation analysis was performed between the expression data of the miRNA transcripts selected in this study and such patients’ characteristics as age, body mass index, and morphology test results (hematocrit and counts of red blood cells, leukocytes, neutrophils, lymphocytes, monocytes, eosinophils, basophils, and platelets), as well as hemoglobin, creatinine, and urea levels. There were no strong or moderate correlations, and only a weak negative correlation (0.4 < |R| < 0.5) was determined between age and expression of has-miR-31-3p (R = −0.42, Benjamini–Hochberg FDR = 0.00026). All correlation results are presented in Table S7.

The two-sided Mann–Whitney *U* test was used to identify relationships between the expression of selected miRNA transcripts and the following categorical variables: sex; smoking (never and former smokers vs. current smokers); hypertension; type 2 diabetes; coronary artery disease; myocardial infarction; and medication with statins, acetylsalicylic acid, clopidogrel, beta-adrenergic blockers, angiotensin-converting enzyme inhibitors, Ca^2+^ channel blockers, fibrates, and micronized diosmin, as well as preparation with hesperidin, *Ruscus aculeatus*, and vitamin C (refer to Table 1). The relationships revealed with a statistical significance of *p* < 0.05 corrected by the Benjamini–Hochberg FDR are presented in Table 3. Six miRNAs (hsa-miR-6503-3p, -4433b-5p, 4433a-3p, -199b-5p, -21-3p, and -1261) were related to sex, four miRNAs (hsa-miR-664a-3p, -199b-5p, -6503-3p, and -21-3p) to medication with acetylsalicylic acid status, and two miRNAs (hsa-miR-664a-3p and -6503-3p) to the medication with diosmin (Table 3, Figure S9). All associated miRNAs were selected from the AAA vs. CVD comparison.

The results of the correlation analysis and Mann–Whitney *U* test showed that certain characteristics could confound differential expression of selected miRNAs. To explore these associations in more detail, multivariate linear regression models were constructed using the expression of particular miRNAs as a dependent variable and all 29 analyzed characteristics as independent variables. The entire results are provided in Table S9. The adjusted coefficients of determination obtained for the performed models were generally low and ranged from −0.059 to 0.351. Furthermore, a low number of variables (maximum 2) in particular models was found to contribute to the explanation of miRNA expression with a statistical significance of *p* < 0.01. These variables, together with associated miRNAs, are presented in Table 4. The characteristics that appeared the most frequently as related to the analyzed miRNAs were monocyte counts and levels of creatinine and urea (Table 4). 

Obtained results indicate that the dysregulations of some miRNAs selected in this study could be affected by differences in demographic and clinical characteristics between the LEAD, AAA, and CVD groups. Although the found relationships seem rather weak, further investigations with larger and more balanced populations are needed to validate these findings.

## 4. Discussion

The elucidation of the molecular mechanisms regulating the pathological conditions responsible for the development of diseases could provide valuable information that could be useful in developing new methods for the management of these diseases. Many recent studies have shown that miRNAs are promising targets for the diagnosis and treatment of various conditions, including LEAD [49,50,51,52,53,54], AAA [28,55,56,57], and CVD [58,59]. In particular, many investigations were carried out to evaluate the utility of proangiogenic properties of miRNAs in the therapy of peripheral atherosclerosis [52]. For example, adipose tissue mesenchymal stem cells transfected with miRNA-126 exhibit a promising therapeutic potential in the diabetic mouse model with critical limb ischemia [49]. The proangiogenic properties of miR-548j-5p have been demonstrated to exert a beneficial effect on hindlimb ischemia in mouse models [50]. Many miRNAs have also been identified to be involved in various types of cardiomyocyte death, making them interesting therapeutic targets in ischemic events such as acute myocardial infarction and heart failure [60].

Our study contributes to the efforts by investigating differences and similarities in miRNA regulatory networks in PBMCs between these diseases. As a result, 10, 8, and 17 differentially expressed miRNA transcripts were selected from LEAD vs. AAA, LEAD vs. CVD, and AAA vs. CVD comparisons, respectively (Figure 1, Table 2).

In our study, a higher expression of hsa-miR-135b-5p was found in the LEAD group compared to patients with AAA and CVD using next-generation sequencing. This result is consistent with a previous study, which demonstrated that this miRNA is upregulated in serum samples from patients with coronary artery disease compared to healthy donors. Further analyses showed that hsa-miR-135b-5p promotes endothelial and vascular smooth muscle cell proliferation by suppressing *MEF2C* expression [61]. Our results suggest that this mechanism could be especially pronounced in LEAD in contrast to AAA and CVD; however, further studies are required to validate this conclusion.

Six miRNAs were found in our study to be dysregulated in arterial diseases (LEAD and AAA) in comparison with CVD (Figure 1). Among them, hsa-miR-99a-5p and hsa-miR-99a-3p were downregulated, and lower expression of both miRNAs was previously associated with the regulation of cell proliferation. hsa-miR-99a-5p was shown to target *HOXA1*, which promotes the proliferation, migration, and invasion of human aortic smooth muscle cells [62]. In turn, hsa-miR-99a-3p was downregulated in head and neck squamous cell carcinoma, and *VEGFA* was reported to be the main target gene for hsa-miR-99a-3p [63]. Therefore, our results showed that the enhancement of cell proliferation resulting from a lower expression of these miRNAs seems to be a common mechanism involved in both LEAD and AAA. This hypothesis could be supported by the upregulation of another miRNA in LEAD and AAA, hsa-miR-196a-5p, which exerts pro-oncogenic properties in various types of cancers [64,65,66].

Furthermore, a higher expression of this miRNA presented in our study was associated with its target, *TSC2* (TSC complex subunit 2), which had a lower expression level in the LEAD group compared to CVD patients (Figure 3). This could indicate a higher proliferation status of LEAD since *TSC2* is a known tumor suppressor acting mainly by inhibiting mTOR signaling [67,68].

Upregulation of hsa-miR-196a-5p was also linked to its other target, *MALT1* (MALT1 paracaspase), which was downregulated in AAA vs. CVD group (Figure 3). Malt1 belongs to CARMA/Bcl10/MALT1 signalosomes activating NF-κB signaling in endothelial and vascular smooth muscle cells and thus promoting vascular inflammation [69,70]. Therefore, this pro-inflammatory mechanism appeared to be more active in CVD than LEAD, and more research is needed to confirm this conclusion.

Our study showed that the increase in expression of hsa-miR-212-3p is accompanied by an elevated level of its potential target, *EIF3C* (eukaryotic translation initiation factor 3 subunit C), in LEAD patients compared to CVD subjects (Figure 3). Increased expression of *EIF3C*, both in cells and exosomes, has previously been shown to promote the development and progression of hepatocellular carcinoma by improving cell proliferation and angiogenesis [71,72]. These processes are also involved in the pathology of atherosclerosis and could be promoted during this disease by upregulation of *EIF3C*.

In our study, a higher level of hsa-miR-124-3p was found in the LEAD group compared to AAA patients. This finding is in line with a previous study, where the expression of this miRNA was increased either in the ischemic tissue of the hindlimb ischemia model or in hypoxic human umbilical vein endothelial cells, where it exerted an anti-angiogenesis effect by targeting *STAT3* [73]. Furthermore, our results showed that the target for this miRNA, *GIT2*, had a higher expression in the AAA group compared to LEAD patients (Figure 3). *GIT2* is a key regulator of aging [74]; therefore, it could be assumed that AAA development is more closely associated with *GIT2*-related aging than LEAD.

Another gene found in our study as a potential target for has-miR-124-3p is *YBX1* (Figure 3). Similarly to *GIT2*, the expression of *YBX1* was found to be higher in AAA patients compared to the LEAD group. This gene has previously been reported to play an important role in aneurysm development through inhibition of proliferation and aggravating apoptosis of smooth muscle cells via upregulation of p21 [75].

These findings suggest that altered expression of hsa-miR-124-3p could change the character of vascular cells between the pro-apoptotic (in AAA) and pro-proliferative state (in LEAD). This hypothesis requires validation in further studies.

In our study, dysregulations in miRNA regulatory networks associated with LEAD, AAA, and CVD were identified to at least partially explain similarities and differences in the molecular mechanisms underlying the pathology of these diseases. However, we would like to emphasize that this work has a descriptive character, and all conclusions presented in Section 4 are hypotheses that should be validated in further studies. Further investigations are needed to evaluate the distribution of miRNA expression in subpopulations of PBMCs and to validate the presented miRNA–gene interactions. The translation potential of the results obtained in PBMCs to other blood components, including serum or plasma, should also be evaluated. It should also be emphasized that the presented study concerns diseases in a specific stage of development. Patients with LEAD suffered intermittent claudication, but without events of critical limb ischemia in the medical history. In the AAA group, patients with aneurysms sized 5.6 to 7.8 cm were included, which are typically classified as large aneurysms, in contrast to small aneurysms characterized by a diameter below 5.5 cm. Regarding CVD, patients with superficial varicose veins with primary etiology and reflux pathophysiology were qualified, without more advanced symptoms, such as edema, skin changes, and ulcers. Furthermore, due to the inability to use qPCR validation and an independent cohort to validate differentially expressed miRNAs in this study, the extended methodology was applied, including DESeq2 and ROC analyses, with multiple thresholds for miRNA selection (*p*-value < 0.05, absolute log2 fold change > 2, and mean of normalized counts > 10). Although the unification of the cut-off criteria for the selection of differentially expressed miRNAs from performed comparisons guaranteed the comparability of the obtained results between the studied groups, it could lead to the loss of some relevant miRNAs. Therefore, further studies focused on genes and miRNAs regulating particular processes closely related to the studied diseases (e.g., angiogenesis or inflammation) should be performed to reveal possible all miRNAs and genes involved in the development of LEAD, AAA, and CVD. Potential bias of our results could also be introduced by demographic and clinical differences between the studied groups. In particular, CVD patients are significantly younger than subjects with other diseases, and hypertensive patients are more represented in the LEAD group than in other groups. There are also clear differences in the medications taken by the studied patients (Table 1). To address this bias, we performed multivariate DESeq2 analysis, and the relationships between such characteristics and dysregulated miRNAs selected in this study were investigated. Certain miRNAs were found to be associated with age, sex, monocyte levels, medication, and other features (Table 3 and Table 4). Therefore, the change in expression of these miRNAs could be affected by these characteristics, and more studies should be performed in larger and more balanced cohorts to validate our results.

Our study demonstrated that investigations of miRNA and whole transcriptome expression profiles provide abundant information about molecular mechanisms that contribute to vascular diseases. The integrated analysis of miRNA and gene expression allowed us to identify dysregulations in the miRNA regulatory network in LEAD, AAA, and CVD. However, more explanatory studies are required to verify the hypotheses drawn from the obtained results and to evaluate the potential diagnostic and therapeutic utility of dysregulated miRNAs and genes presented in this study.

## 5. Conclusions

The presented work demonstrated similarities and differences in the dysregulations of the miRNA regulatory network in LEAD, AAA, and CVD. The identified miRNAs and their potential targets revealed abundant information about vascular pathology, providing many hypotheses on how dysregulated miRNA function in that PBMCs can contribute to the development of LEAD, AAA, and CVD. Therefore, this study indicates new research paths for further validation studies and provides new candidates for transcriptomic biomarkers of LEAD, AAA, and CVD with potential utility in differentiating these diseases.

## Figures and Tables

**Table 1:** Demographical and clinical information regarding the study subjects. The table presents extended data gathered from our previous studies [40,41,42].

Characteristic	Patients with LEAD (*n* = 40)	Patients with AAA (*n* = 28)	Patients with CVD (*n* = 34)	*p* ^1^
Age	57.6 ± 9.82 56.5 (43–71)	66.4 ± 4.52 67 (57–76)	44.1 ± 10.07 42 (27–78)	9.966 × 10^−12^
Sex males/females	35 (87.5%)/5 (12.5%)	25 (89.3%)/ 3 (10.7%)	17 (50%)/ 17 (50%)	2.180 × 10^−4^
Body mass index (BMI)	27.2 ± 2.62 27.3 (21.9–31.6)	25.1 ± 3.30 25.2 (18.0–31.3)	23.9 ± 2.35 24.0 (20.1–28.8)	1.670 × 10^−5^
**Risk factors and cardiovascular comorbidities**				
Smoking never/former/current	0 (0%)/18 (45%)/ 22 (55%)	9 (32.1%)/ 9 (32.1%)/10 (35.7%)	16 (47%)/13 (38%)/ 5 (14.7%)	2.369 × 10^−5^
Diabetes type 2	5 (12.5%)	6 (21.4%)	0 (0%)	9.309 × 10^−3^
Hypertension	36 (90%)	19 (67.9%)	0 (0%)	1.066 × 10^−17^
Coronary artery disease (CAD)	11 (27.5%)	7 (25%)	0 (0%)	8.508 × 10^−4^
Myocardial infarction	8 (20%)	3 (10.7%)	0 (0%)	0.011
Stroke/transient ischemic attack	2 (5%)	1 (3.6%)	0 (0%)	0.493
**Hematological and biochemical blood parameters**				
Red blood cells (M/µL)	4.74 ± 0.30 4.75 (4.11–5.18)	4.94 ± 0.21 4.99 (4.56–5.50)	4.78 ± 0.34 4.83 (3.78–5.50)	0.017
White blood cells (K/µL)	5.49 ± 0.69 5.27 (4.45–6.89)	5.66 ± 0.70 5.78 (4.44–6.90)	5.76 ± 0.71 5.78 (4.67–6.90)	0.361
Lymphocytes (K/µL)	3.04 ± 0.54 3.03 (2.01–3.99)	2.99 ± 0.44 3.02 (2.05–3.78)	2.99 ± 0.45 3.00 (2.04–3.99)	0.771
Monocytes (K/µL)	0.47 ± 0.15 0.41 (0.22–0.87)	0.39 ± 0.12 0.33 (0.26–0.67)	0.33 ± 0.07 0.32 (0.19–0.56)	9.727 × 10^−6^
Neutrophils (K/µL)	4.21 ± 0.47 4.07 (3.51–5.21)	4.17 ± 0.43 4.20 (3.33–4.78)	4.17 ± 0.62 4.31 (2.12–4.99)	0.809
Eosinophils (K/µL)	0.21 ± 0.09 0.20 (0.10–0.56)	0.18 ± 0.05 0.18 (0.11–0.31)	0.16 ± 0.04 0.17 (0.08–0.23)	6.072 × 10^−3^
Basophils (K/µL)	0.10 ± 0.03 0.1 (0.07–0.19)	0.11 ± 0.02 0.11 (0.08–0.20)	0.10 ± 0.03 0.10 (0.02–0.20)	3.955 × 10^−3^
Platelets (K/µL)	309.3 ± 75.7 295.5 (179–561)	419.9 ± 124.0 404.5 (211–756)	368.26 ± 104.15 365.5 (211–756)	4.643 × 10^−5^
Hemoglobin (g/dL)	14.12 ± 0.52 14.01 (12.99–14.99)	14.02 ± 0.51 13.99 (13.34–15.00)	14.08 ± 0.48 14.06 (13.30–14.99)	0.692
Hematocrit (%)	41.3 ± 1.42 41.5 (38.4–43.8)	40.8 ± 1.30 41 (38–43)	39.9 ± 1.63 40 (37–43)	6.775 × 10^−4^
Creatinine (mmol/L)	78.7 ± 12.6 79 (56–99)	54.2 ± 11.5 51.5 (39–87)	58.2 ± 14.20 56 (37–93)	2.086 × 10^−10^
Urea (mmol/L)	4.69 ± 0.83 4.79 (2.99–6.02)	4.66 ± 0.67 4.69 (3.45–5.88)	5.09 ± 0.86 4.94 (3.45–6.87)	0.119
**Medication**				
Statins	34 (85%)	13 (46.4%)	0 (0%)	2.090 × 10^−13^
Acetylsalicylic acid	40 (100%)	27 (96.4%)	0 (0%)	7.961 × 10^−26^
Clopidogrel	8 (20%)	3 (10.7%)	0 (0%)	6.382 × 10^−3^
Beta-adrenergic blockers	27 (67.5%)	16 (57.1%)	0 (0%)	4.427 × 10^−12^
Angiotensin-converting enzyme inhibitor	20 (50%)	4 (14.3%)	0 (0%)	6.266 × 10^−7^
Ca^2+^ channel blockers	11 (27.5%)	2 (7.14%)	0 (0%)	5.431 × 10^−4^
Fibrates	5 (12.5%)	2 (7.14%)	0 (0%)	0.092
Metformin	2 (5%)	3 (10.7%)	0 (0%)	0.148
Gliclazide	4 (10%)	4 (14.3%)	0 (0%)	0.055
Micronized diosmin	0 (0%)	0 (0%)	24 (70.6%)	1.012 × 10^−15^
Preparation with hesperidin, *Ruscus aculeatus*, and vitamin C	0 (0%)	0 (0%)	15 (44.1%)	4.550 × 10^−8^

^1^ Statistical significance of differences between lower extremity artery disease (LEAD), abdominal aortic aneurysm (AAA), and chronic venous disease (CVD) groups, calculated using Kruskal–Wallis rank sum test for continuous-type variables (age, BMI, hematological and biochemical blood parameters) and two-sided Fisher’s exact test for categorical-type variables (sex, risk factors, cardiovascular comorbidities, and medication). Data for continuous-type variables were expressed as mean ± SD and median (range). Data for categorical-type variables were expressed as numbers (percentage).

**Table 2:** Differential expression parameters of 10, 8, and 17 miRNA transcripts selected from LEAD vs. AAA, LEAD vs. CVD, and AAA vs. CVD comparisons, respectively.

No.	miRNA Transcript	miRNA ID ^1^	*p*	Fold Change	ROC-AUC
LEAD vs. AAA—Upregulated miRNAs					
1.	hsa-mir-124-2_hsa-miR-124-3p	hsa-miR-124-3p	6.715 × 10^−4^	7.441	0.585
2.	hsa-mir-124-1_hsa-miR-124-3p	hsa-miR-124-3p	1.665 × 10^−3^	6.015	0.550
3.	hsa-mir-124-3_hsa-miR-124-3p	hsa-miR-124-3p	1.946 × 10^−3^	6.455	0.562
4.	hsa-mir-7641-2_hsa-miR-7641	hsa-miR-7641	7.019 × 10^−3^	3.398	0.646
5.	hsa-mir-205_hsa-miR-205-5p	hsa-miR-205-5p	9.943 × 10^−3^	3.414	0.558
6.	hsa-mir-31_hsa-miR-31-3p	hsa-miR-31-3p	1.066 × 10^−2^	2.040	0.749
7.	hsa-mir-135b_hsa-miR-135b-5p	hsa-miR-135b-5p	1.121 × 10^−2^	2.565	0.656
8.	hsa-mir-7641-1_hsa-miR-7641	hsa-miR-7641	2.886 × 10^−2^	2.231	0.629
**LEAD vs. AAA—Downregulated miRNAs**					
9.	hsa-mir-4531_hsa-miR-4531	hsa-miR-4531	8.039 × 10^−4^	0.439	0.521
10.	hsa-mir-1303_hsa-miR-1303	hsa-miR-1303	9.764 × 10^−4^	0.386	0.629
**LEAD vs. CVD—Upregulated miRNAs**					
1.	hsa-mir-196a-1_hsa-miR-196a-5p	hsa-miR-196a-5p	7.766 × 10^−3^	14.106	0.603
2.	hsa-mir-98_hsa-miR-98-5p	hsa-miR-98-5p	1.599 × 10^−2^	2.086	0.646
3.	hsa-mir-212_hsa-miR-212-3p	hsa-miR-212-3p	3.163 × 10^−2^	2.339	0.558
4.	hsa-mir-135b_hsa-miR-135b-5p	hsa-miR-135b-5p	3.597 × 10^−2^	7.939	0.524
**LEAD vs. CVD—Downregulated miRNAs**					
5.	hsa-mir-99a_hsa-miR-99a-5p	hsa-miR-99a-5p	5.074 × 10^−3^	0.211	0.626
6.	hsa-mir-99a_hsa-miR-99a-3p	hsa-miR-99a-3p	5.534 × 10^−3^	0.113	0.607
7.	hsa-mir-125b-2_hsa-miR-125b-5p	hsa-miR-125b-5p	1.317 × 10^−2^	0.259	0.659
8.	hsa-mir-193b_hsa-miR-193b-3p	hsa-miR-193b-3p	4.348 × 10^−2^	0.263	0.527
**AAA vs. CVD—Upregulated miRNAs**					
1.	hsa-mir-98_hsa-miR-98-5p	hsa-miR-98-5p	5.135 × 10^−3^	2.227	0.674
2.	hsa-mir-196a-1_hsa-miR-196a-5p	hsa-miR-196a-5p	8.853 × 10^−3^	11.438	0.516
3.	hsa-mir-199b_hsa-miR-199b-5p	hsa-miR-199b-5p	1.133 × 10^−2^	2.608	0.750
4.	hsa-mir-7-1_hsa-miR-7-5p	hsa-miR-7-5p	1.496 × 10^−2^	2.079	0.601
5.	hsa-mir-1261_hsa-miR-1261	hsa-miR-1261	1.766 × 10^−2^	2.256	0.611
6.	hsa-mir-21_hsa-miR-21-3p	hsa-miR-21-3p	3.115 × 10^−2^	2.567	0.798
7.	hsa-mir-99b_hsa-miR-99b-3p	hsa-miR-99b-3p	3.642 × 10^−2^	3.652	0.564
8.	hsa-mir-6503_hsa-miR-6503-3p	hsa-miR-6503-3p	4.350 × 10^−2^	2.313	0.733
9.	hsa-mir-550a-3_hsa-miR-550a-3p	hsa-miR-550a-3p	4.373 × 10^−2^	2.026	0.693
**AAA vs. CVD—Downregulated miRNAs**					
10.	hsa-mir-664a_hsa-miR-664a-3p	hsa-miR-664a-3p	1.218 × 10^−4^	0.499	0.824
11.	hsa-mir-125b-2_hsa-miR-125b-5p	hsa-miR-125b-5p	8.773 × 10^−4^	0.182	0.619
12.	hsa-mir-99a_hsa-miR-99a-5p	hsa-miR-99a-5p	1.189 × 10^−3^	0.185	0.608
13.	hsa-mir-99a_hsa-miR-99a-3p	hsa-miR-99a-3p	1.251 × 10^−3^	0.092	0.640
14.	hsa-mir-125b-1_hsa-miR-125b-5p	hsa-miR-125b-5p	6.648 × 10^−3^	0.247	0.598
15.	hsa-mir-4433b_hsa-miR-4433b-5p	hsa-miR-4433b-5p	2.297 × 10^−2^	0.197	0.544
16.	hsa-mir-4433_hsa-miR-4433-3p	hsa-miR-4433a-3p	2.297 × 10^−2^	0.197	0.544
17.	hsa-mir-193b_hsa-miR-193b-3p	hsa-miR-193b-3p	4.728 × 10^−2^	0.292	0.450

^1^ According to miRBase 22.1 (http://www.mirbase.org). The table presents *p* and fold change values received from the DESeq2 analysis and areas under ROC curves (ROC-AUC) received from the ROC analysis. miRNA transcripts were divided into upregulated and downregulated groups within each comparison and ordered according to increasing *p*-value. AAA—abdominal aortic aneurysm, CVD—chronic venous disease, LEAD—lower extremity artery disease, ROC—receiver operating characteristic.

**Table 3:** Relationships between categorical characteristics of study subjects and expression of miRNA transcripts selected from LEAD vs. AAA, LEAD vs. CVD, and AAA vs. CVD comparisons. The table presents statistically significant relationships (*p* < 0.05, Benjamini–Hochberg FDR correction) obtained from two-sided Mann–Whitney U test (the entire results are provided in Table S8).

Characteristic	miRNA Transcript	miRNA ID ^1^	*p*
Sex	hsa-mir-6503_hsa-miR-6503-3p	hsa-miR-6503-3p	3.02 × 10^−3^
	hsa-mir-4433b_hsa-miR-4433b-5p	hsa-miR-4433b-5p	1.17 × 10^−2^
	hsa-mir-4433_hsa-miR-4433-3p	hsa-miR-4433a-3p	1.17 × 10^−2^
	hsa-mir-199b_hsa-miR-199b-5p	hsa-miR-199b-5p	1.17 × 10^−2^
	hsa-mir-21_hsa-miR-21-3p	hsa-miR-21-3p	1.48 × 10^−2^
	hsa-mir-1261_hsa-miR-1261	hsa-miR-1261	2.21 × 10^−2^
Acetylsalicylic acid medication	hsa-mir-664a_hsa-miR-664a-3p	hsa-miR-664a-3p	3.02 × 10^−3^
	hsa-mir-199b_hsa-miR-199b-5p	hsa-miR-199b-5p	9.42 × 10^−3^
	hsa-mir-6503_hsa-miR-6503-3p	hsa-miR-6503-3p	1.17 × 10^−2^
	hsa-mir-21_hsa-miR-21-3p	hsa-miR-21-3p	1.48 × 10^−2^
Diosmin medication	hsa-mir-664a_hsa-miR-664a-3p	hsa-miR-664a-3p	3.42 × 10^−2^
	hsa-mir-6503_hsa-miR-6503-3p	hsa-miR-6503-3p	4.04 × 10^−2^

^1^ According to miRBase 22.1 (http://www.mirbase.org).

**Table 4:** Results of the multivariate linear regression performed using the expression of particular miRNA transcripts as a dependent variable and the characteristics of the studied subjects as independent variables. The table presents regression coefficients and associated *p*-values of characteristics resulting in *p* < 0.01. The entire results of the multivariate regression analysis are provided in Table S9.

Characteristic	miRNA Transcript	miRNA ID ^1^	Regression Coefficient	*p*
Monocyte counts	hsa-mir-99b_hsa-miR-99b-3p ^4^	hsa-miR-99b-3p	−1.38	6.07 × 10^−3^
	hsa-mir-4433b_hsa-miR-4433b-5p ^4^	hsa-miR-4433b-5p	−2.12	6.03 × 10^−3^
	hsa-mir-4433_hsa-miR-4433-3p ^4^	hsa-miR-4433a-3p	−2.12	6.03 × 10^−3^
	hsa-mir-31_hsa-miR-31-3p ^2^	hsa-miR-31-3p	2.23	2.01 × 10^−3^
	hsa-mir-7-1_hsa-miR-7-5p ^4^	hsa-miR-7-5p	0.92	1.44 × 10^−3^
Creatinine	hsa-mir-125b-1_hsa-miR-125b-5p ^4^	hsa-miR-125b-5p	−0.01	2.02 × 10^−3^
	hsa-mir-125b-2_hsa-miR-125b-5p ^3,4^	hsa-miR-125b-5p	−0.01	6.17 × 10^−3^
Urea	hsa-mir-125b-1_hsa-miR-125b-5p ^4^	hsa-miR-125b-5p	0.20	5.24 × 10^−3^
	hsa-mir-125b-2_hsa-miR-125b-5p ^3,4^	hsa-miR-125b-5p	0.21	4.55 × 10^−3^
Sex	hsa-mir-7641-2_hsa-miR-7641 ^2^	hsa-miR-7641	0.72	4.40 × 10^−3^
Myocardial infarction	hsa-mir-7-1_hsa-miR-7-5p ^4^	hsa-miR-7-5p	−0.57	5.99 × 10^−3^

^1^ According to miRBase 22.1 (http://www.mirbase.org), ^2^ miRNA transcripts selected from LEAD vs. AAA comparison, ^3^ miRNA transcripts selected from LEAD vs. CVD comparison, ^4^ miRNA transcripts selected from AAA vs. CVD comparison, *p*—statistical significance, LEAD—lower extremity artery disease, AAA—abdominal aortic aneurysm, CVD—chronic venous disease.

## Data Availability

The data generated for this study are openly available in the FigShare repository at https://doi.org/10.6084/m9.figshare.19446851.v2.

## Supplementary Materials

The following supporting information can be downloaded at: https://www.mdpi.com/article/10.3390/jcm11123470/s1, Figure S1: Proportions of white blood cell subpopulations in the groups of patients with LEAD (lower extremity artery disease), AAA (abdominal aortic aneurysm), and CVD (chronic venous disease), Figure S2: Boxplot presenting log10 of Cook’s distances of miRNAs across samples, Figure S3: Quality control plots generated for results of differential miRNA expression analysis performed by DESeq2 package between 40 LEAD and 34 CVD patients, Figure S4: Quality control plots generated for results of differential miRNA expression analysis performed by DESeq2 package between 40 LEAD and 28 AAA patients, Figure S5: Quality control plots generated for results of differential miRNA expression analysis performed by DESeq2 package between 28 AAA and 34 CVD patients, Figure S6: Receiver operating characteristic (ROC) analysis plots performed for 10 miRNA transcripts selected from LEAD vs. AAA comparison, Figure S7: Receiver operating characteristic (ROC) analysis plots performed for 8 miRNA transcripts selected from LEAD vs. CVD comparison, Figure S8: Receiver operating characteristic (ROC) analysis plots performed for 17 miRNA transcripts selected from AAA vs. CVD comparison, Figure S9: Boxplots presenting expression of miRNAs in relation to their statistically significantly associated categorical characteristics of study population, as resulted from two-sided Mann–Whitney U test, Table S1: Differentially expressed miRNAs resulting from LEAD vs. AAA comparison using multivariate DESeq2 analysis, Table S2: Differentially expressed miRNAs resulting from LEAD vs. CVD comparison using multivariate DESeq2 analysis, Table S3: Differentially expressed miRNAs resulting from AAA vs. CVD comparison using multivariate DESeq2 analysis, Table S4: Results of receiver operating characteristic (ROC) analysis performed for 10, 8, and 17 miRNA transcripts selected from LEAD vs. AAA, LEAD vs. CVD, and AAA vs. CVD comparisons, respectively, Table S5: Results of functional analysis performed using miRNet 2.0 website tool (https://www.mirnet.ca) on 7, 8, and 16 miRNAs selected from LEAD vs. AAA, LEAD vs. CVD, and AAA vs. CVD comparisons, respectively, Table S6: Interactions identified between miRNAs selected in this study from LEAD vs. AAA, LEAD vs. CVD, and AAA vs. CVD comparisons and genes selected in our previous study from corresponding comparisons, Table S7: Results of correlation analysis between characteristics of studied groups and expression of 10, 8, and 17 miRNA transcripts selected from LEAD vs. AAA, LEAD vs. CVD, and AAA vs. CVD comparisons, respectively, Table S8: Results of two-sided Mann–Whitney U test performed to investigate differences between categorical characteristics of study subjects and expression of 10, 8, and 17 miRNA transcripts selected from LEAD vs. AAA, LEAD vs. CVD, and AAA vs. CVD comparisons, respectively, Table S9: Results of multivariate regression analysis between characteristics of studied groups (independent variables) and expression of 10, 8, and 17 miRNA transcripts (dependent variables) selected from LEAD vs. AAA, LEAD vs. CVD, and AAA vs. CVD comparisons, respectively.
