# Supplementary material for: miRNA Regulatory Networks Associated with Peripheral Vascular Diseases
Source: J Clin Med. 2022 Jun 16;11(12):3470. doi: 10.3390/jcm11123470 (PMC9224609; doi:10.3390/jcm11123470)
Supplement: Supplementary file 1 [file jcm-11-03470-s001.zip › SupplementaryFigures.pdf]

SUPPLEMENTARY MATERIAL

Supplementary Figures

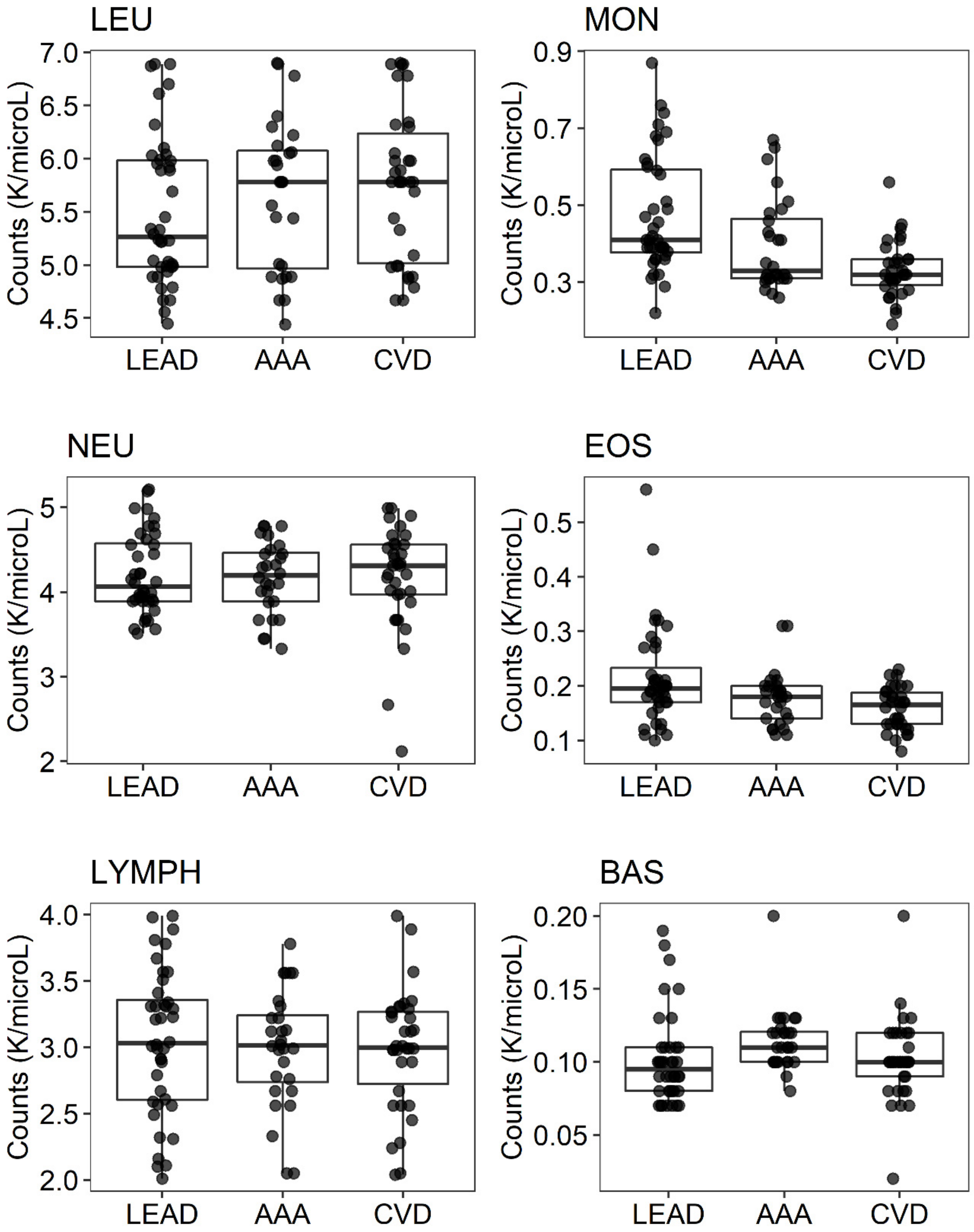

Figure S1. Proportions of white blood cells subpopulations in the groups of patients with LEAD (lower extremity artery disease), AAA (abdominal aortic aneurysm) and CVD (chronic venous disease). Data was collected from blood morphology tests. LEU – leukocytes, NEU – neutrophils, LYMPH – lymphocytes, MON – monocytes, EOS – eosinophils, BAS – basophils.

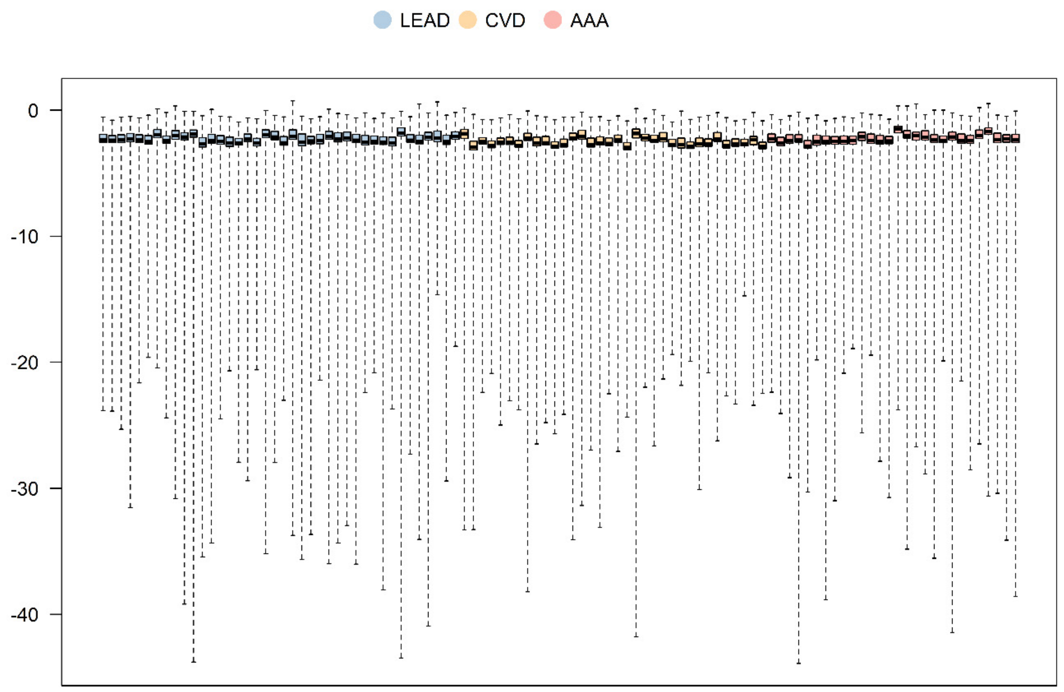

Figure S2. Boxplot presenting log10 of Cook's distances of miRNAs across samples. Whiskers define a range between minimum and maximum value of Cook's distance, boxes range between 25% and 75% quartile, horizontal lines inside boxes mark median value. LEAD - lower extremity artery disease, AAA - abdominal aortic aneurysm, CVD - chronic venous disease.

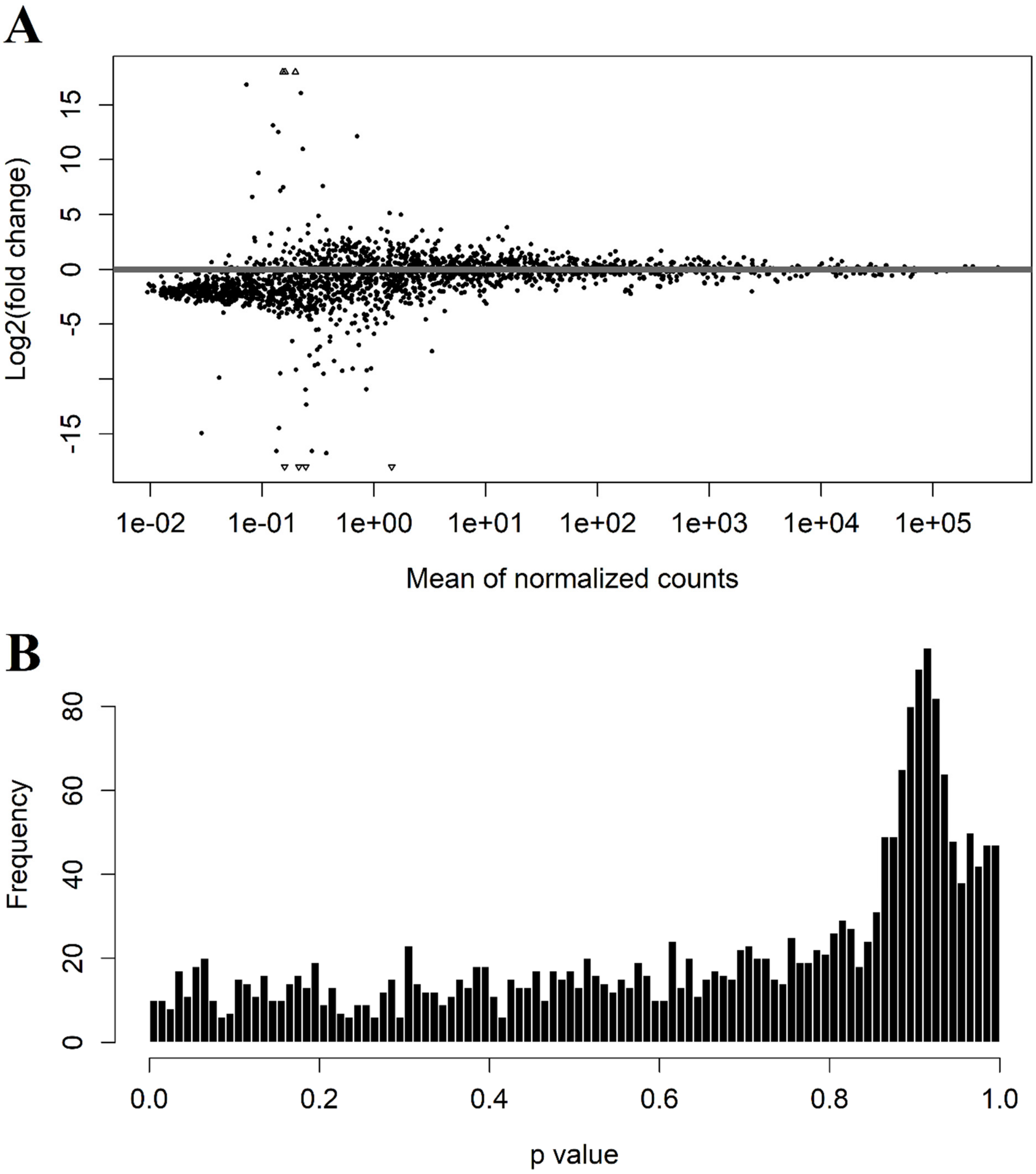

Figure S3. Quality control plots generated for results of differential miRNA expression analysis performed by DESeq2 package between 40 LEAD and 34 CVD patients. (**A**) MA plot showing log2 fold changes of differentially expressed miRNAs against means of normalized counts. (**B**) Distribution of *p* values.

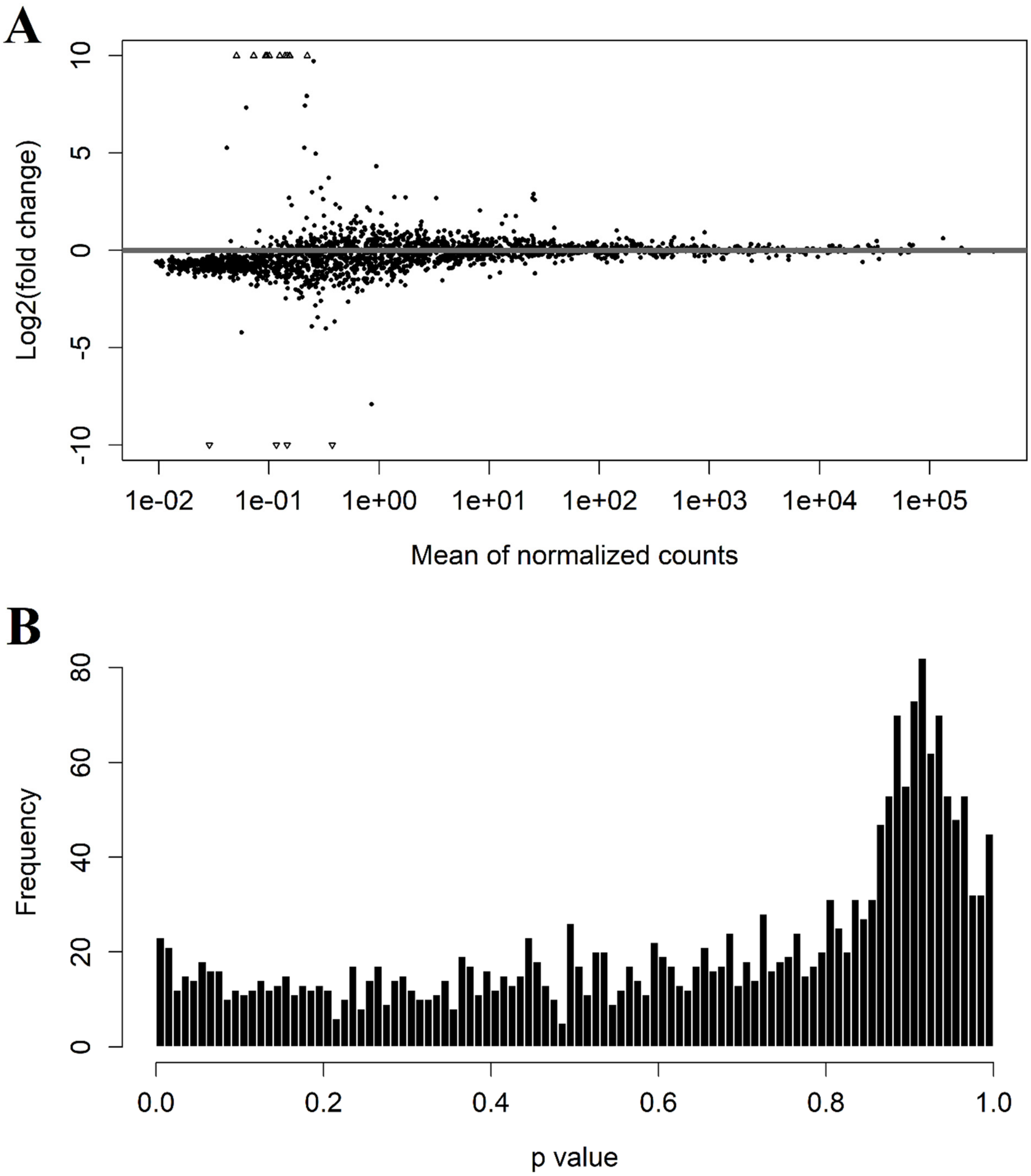

Figure S4. Quality control plots generated for results of differential miRNA expression analysis performed by DESeq2 package between 40 LEAD and 28 AAA patients. (**A**) MA plot showing log2 fold changes of differentially expressed miRNAs against means of normalized counts. (**B**) Distribution of *p* values.

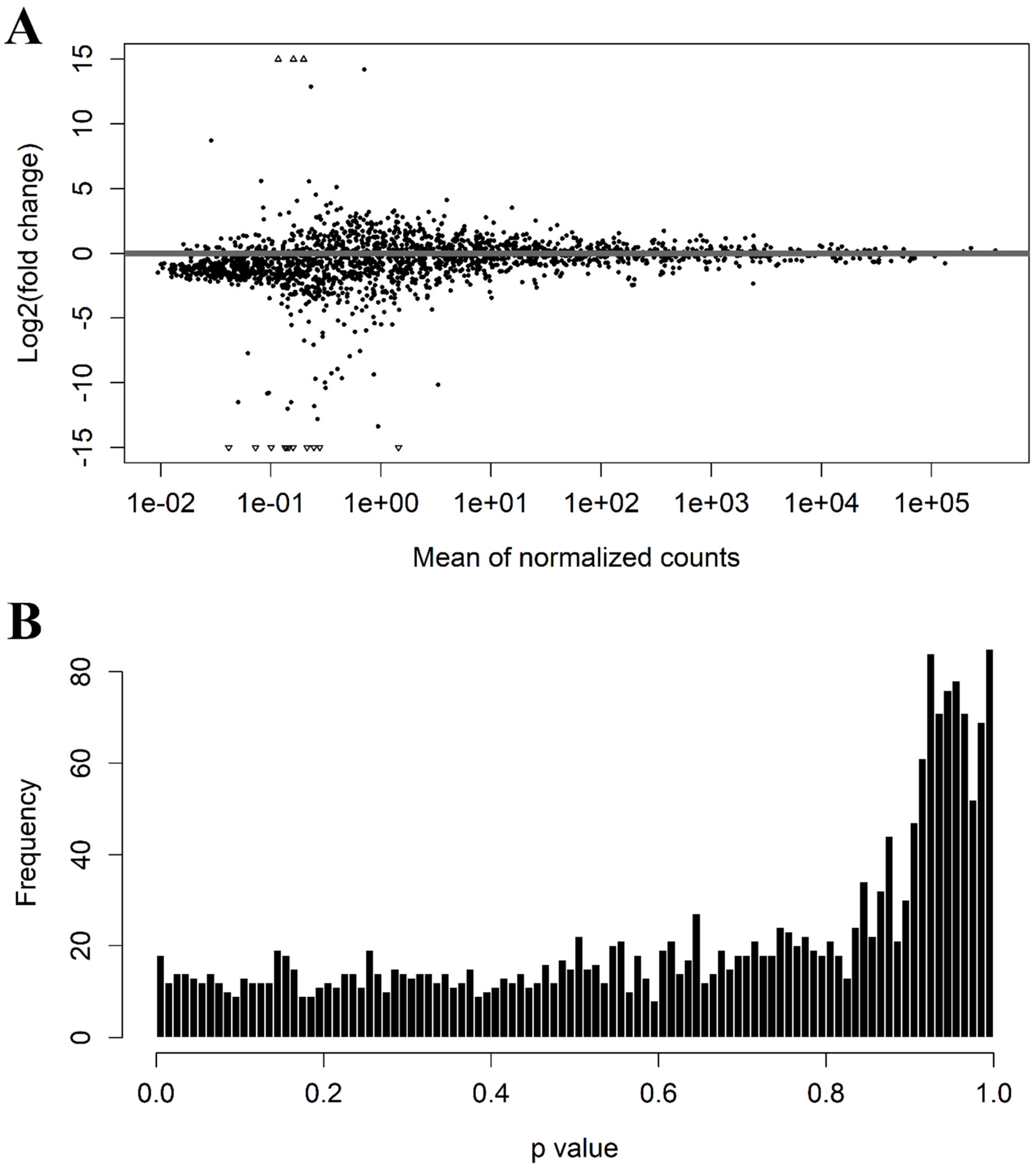

Figure S5. Quality control plots generated for results of differential miRNA expression analysis performed by DESeq2 package between 28 AAA and 34 CVD patients. (**A**) MA plot showing log2 fold changes of differentially expressed miRNAs against means of normalized counts. (**B**) Distribution of *p* values.

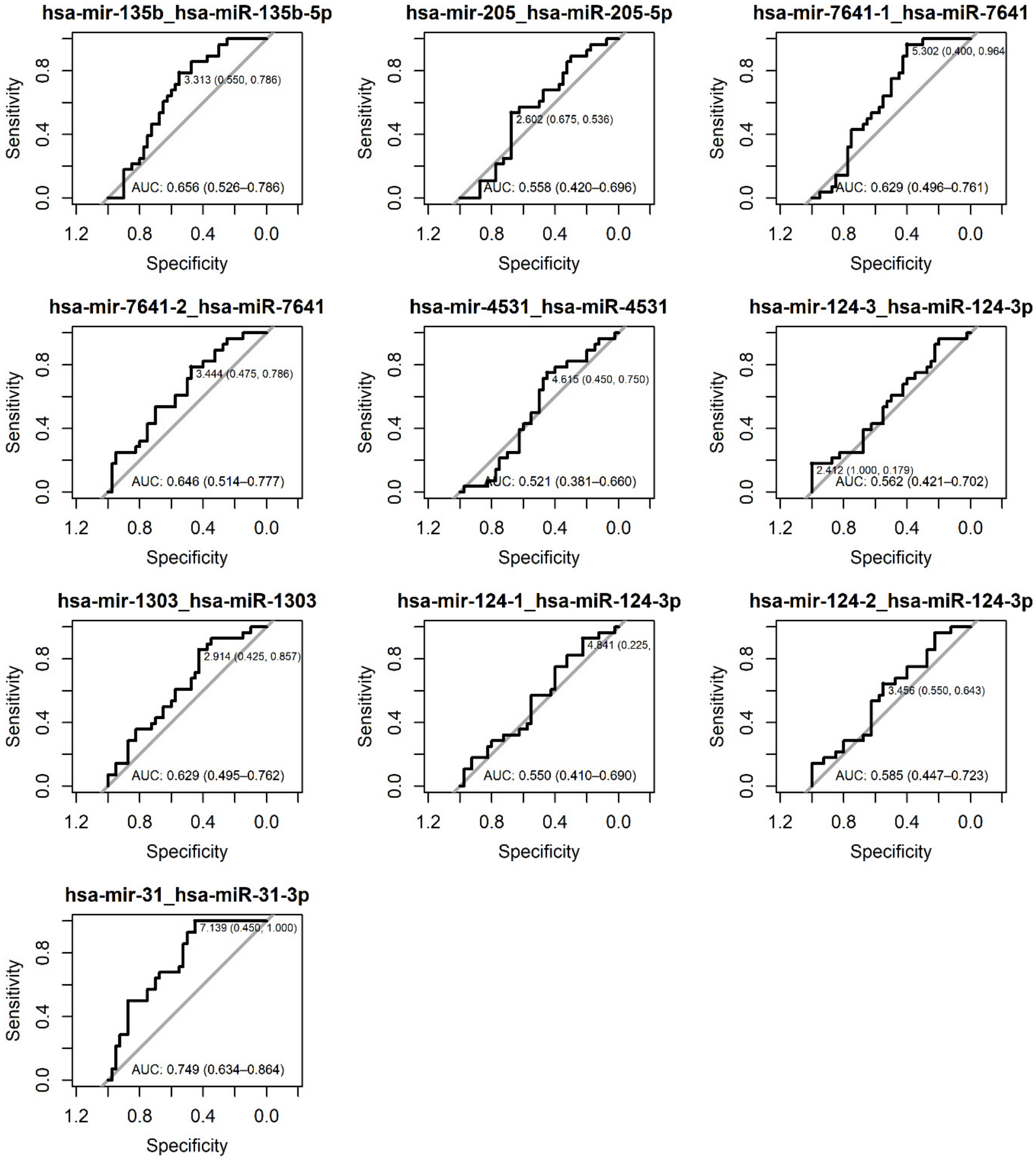

Figure S6. Receiver Operating Characteristics (ROC) analysis plots performed for 10 miRNA transcripts selected from LEAD vs. AAA comparison. LEAD - lower extremity artery disease, AAA - abdominal aortic aneurysm.

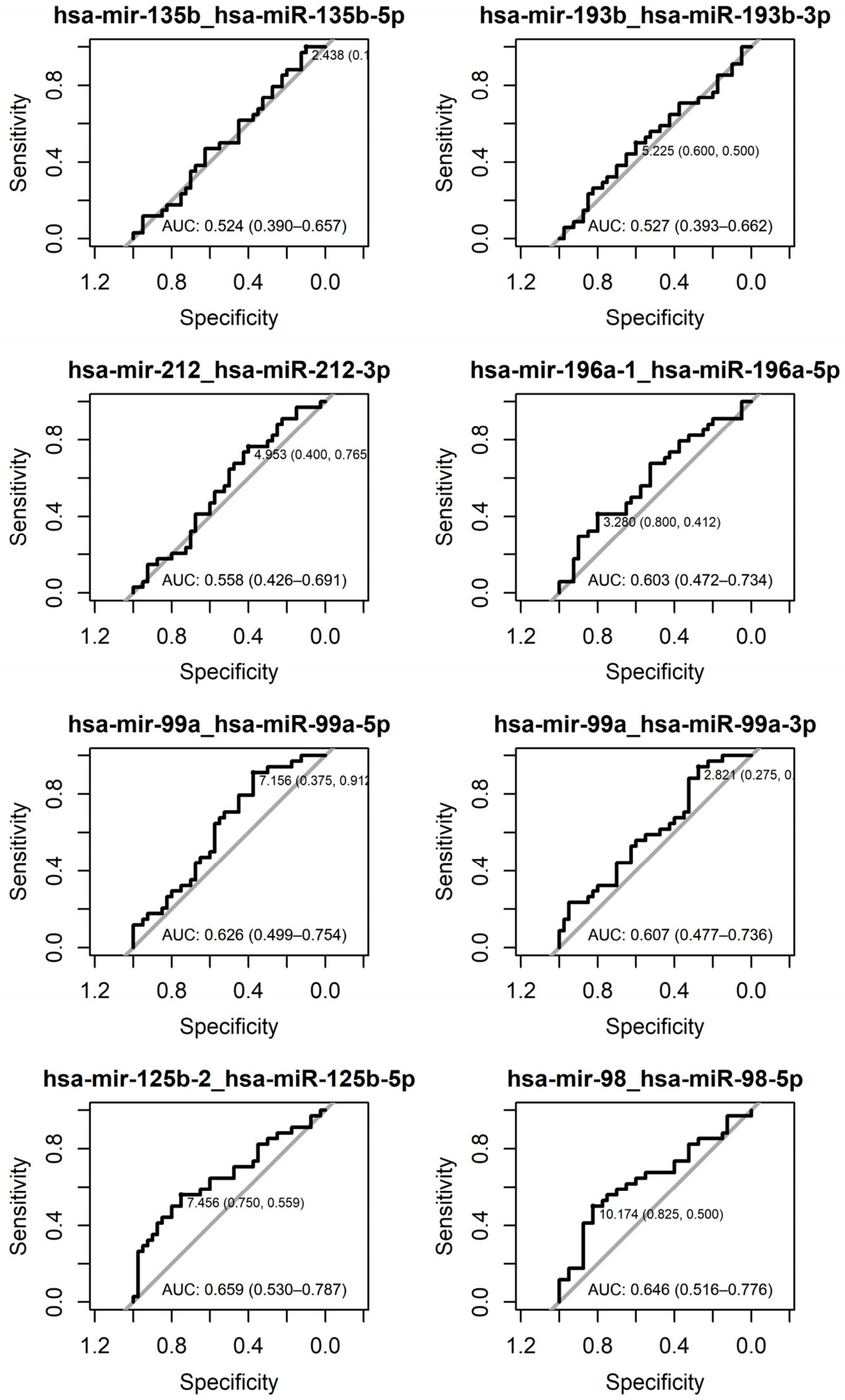

Figure S7. Receiver Operating Characteristics (ROC) analysis plots performed for 8 miRNA transcripts selected from LEAD vs. CVD comparison. LEAD - lower extremity artery, CVD - chronic venous disease.

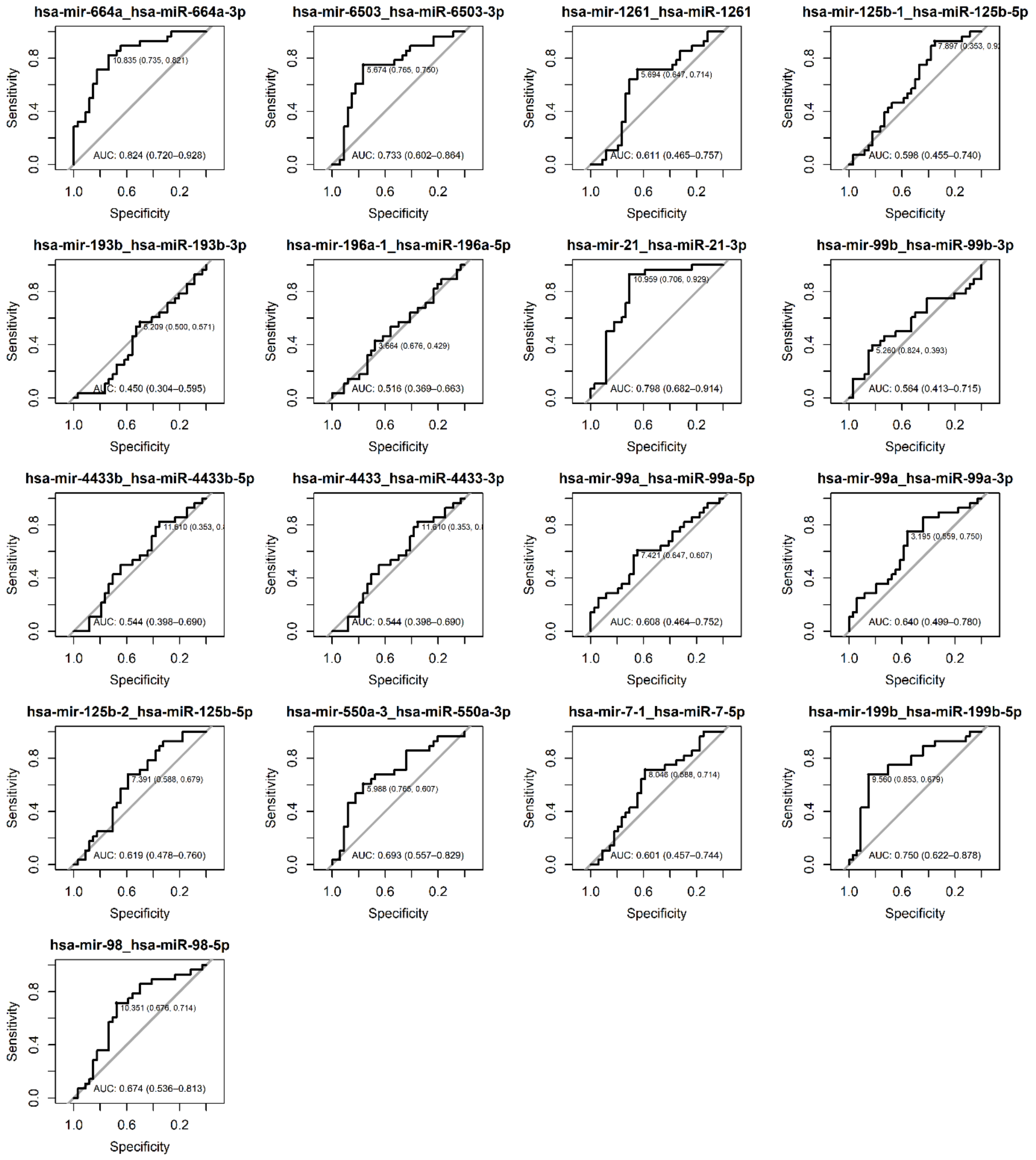

Figure S8. Receiver Operating Characteristics (ROC) analysis plots performed for 17 miRNA transcripts selected from AAA vs. CVD comparison. AAA - abdominal aortic aneurysm, CVD - chronic venous disease.

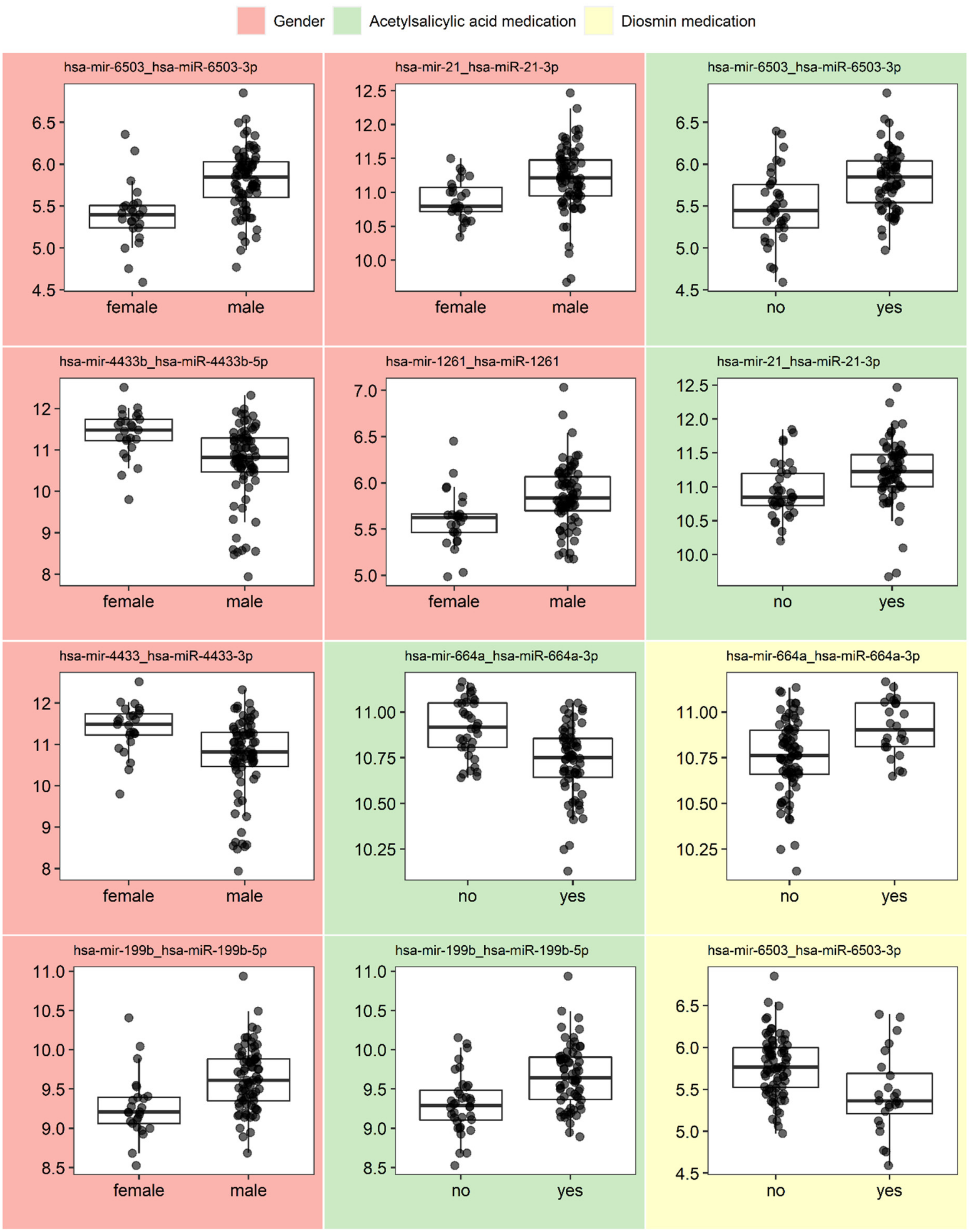

Figure S9. Boxplots presenting expression of miRNAs in relation to their statistically significantly associated categorical characteristics of study population, as resulted from two-sided Mann–Whitney *U* test.
